# Author Correction: Ultra-bright Raman dots for multiplexed optical imaging

**DOI:** 10.1038/s41467-021-22585-3

**Published:** 2021-04-01

**Authors:** Zhilun Zhao, Chen Chen, Shixuan Wei, Hanqing Xiong, Fanghao Hu, Yupeng Miao, Tianwei Jin, Wei Min

**Affiliations:** 1 Department of Chemistry, Columbia University, New York, NY USA; 2 Department of Applied Physics and Applied Mathematics, Columbia University, New York, NY USA

**Keywords:** Optical imaging, Nanoparticles, Raman spectroscopy

Correction to: *Nature Communications* 10.1038/s41467-021-21570-0, published online 26 February 2021.

The original version of this Article omitted Zhilun Zhao and Chen Chen as equally contributing authors. This has now been corrected in both the PDF and HTML versions of the Article.

