# Supplementary material for: Ultra-bright Raman dots for multiplexed optical imaging
Source: Nat Commun. 2021 Feb 26;12:1305. doi: 10.1038/s41467-021-21570-0 (PMC7910594; doi:10.1038/s41467-021-21570-0)
Supplement: Supplementary file 1 — Supplementary Information [file 41467_2021_21570_MOESM1_ESM.pdf]

Ultra-bright Raman dots (Rdots) for multiplexed optical imaging

Zhilun Zhao[1], Chen Chen[1], Shixuan Wei[1], Hanqing Xiong[1], Fanghao Hu[1], Yupeng Miao[1], Tianwei Jin[2] and Wei Min[1]*

[1] Department of Chemistry, Columbia University, New York, NY 10027

[2] Department of Applied Physics and Applied Mathematics, Columbia University, New York, NY 10027

*Corresponding author: wm2256@columbia.edu

Supplementary Information

Supplementary figures

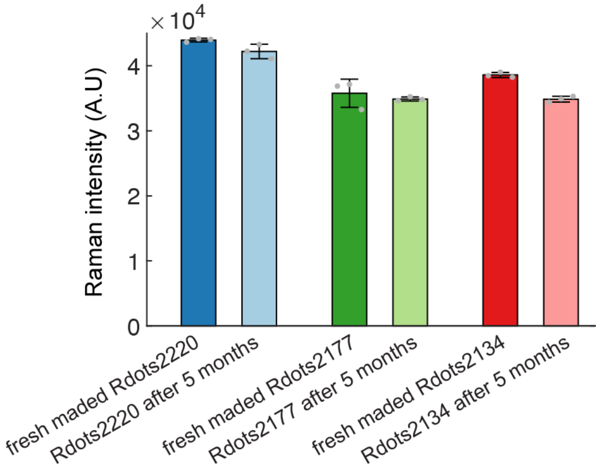

Supplementary Figure 1. Stability of Rdots. The intensities of three kinds of Rdots were measured when they were made and after 5 months in aqueous solution. No signal decrease was observed compared to fleshly made Rdots. Error bars indicate mean ± s.d, n = 3. Raman intensities were normalized to Rdots concentration. A.U.: arbitrary units.

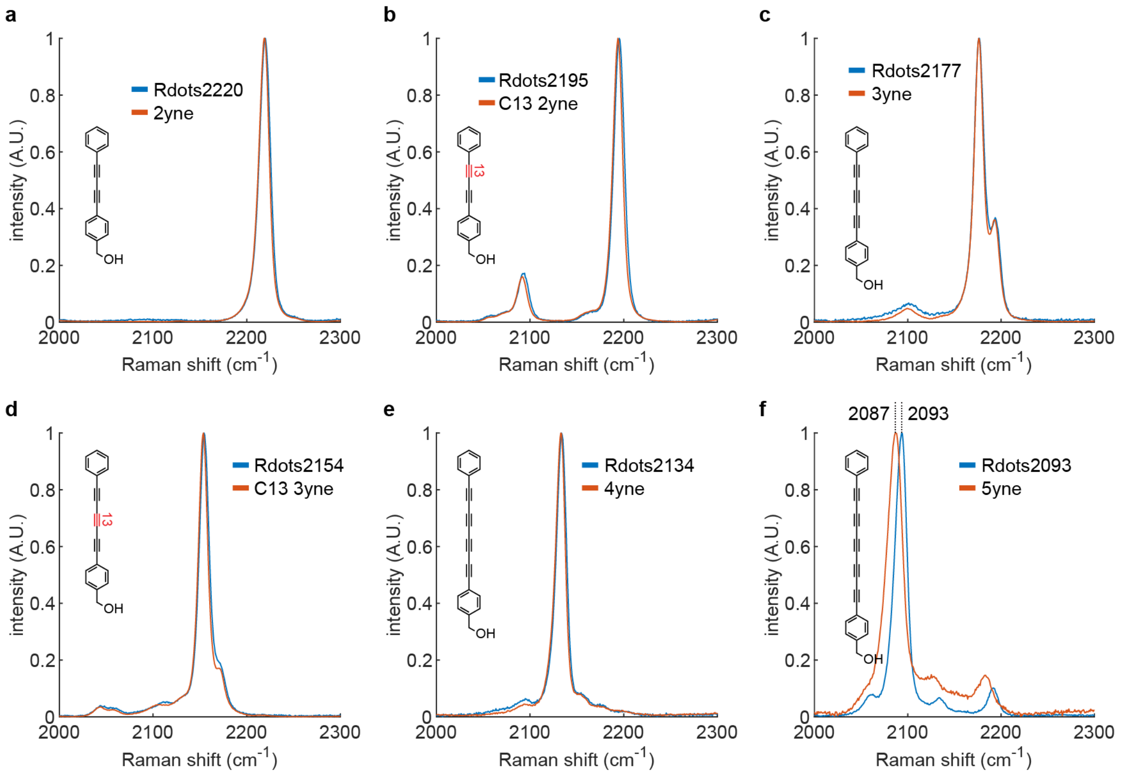

Supplementary Figure 2. Raman spectra of free dyes and corresponding Rdots. The Raman spectra were acquired for free Carbow dyes dissolved in DMSO and Rdots respectively. Most Carbow dyes did not show spectral difference. For 5-yne **(f)**, a red shift around 6 cm^{-1} was observed, but not the spectral broadening. This is likely due to the hydrophobicity of the environment. DMSO is more polar than other solvent such as toluene which should have similar polarity as the PS nanoparticle matrix. It was known that for some molecules, the alkyne vibration frequency can be red shifted by polar solvent such as DMSO[1]. A.U.: arbitrary units.

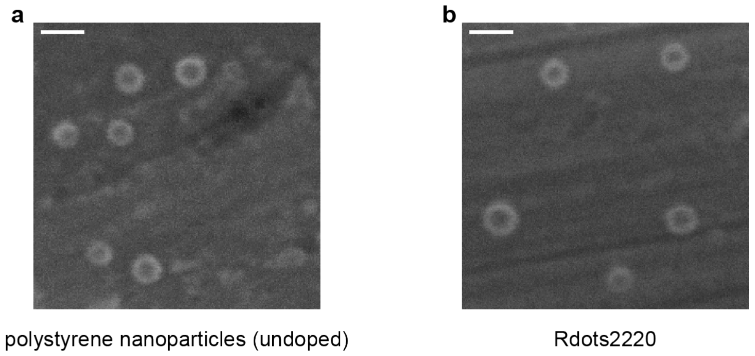

Supplementary Figure 3. Unchanged size and morphology before and after Raman dye doping. The SEM images shows that compared to the undoped polystyrene nanoparticles (a), the Raman dye doped Rdots2220 (b) have similar size and morphology. Scale bar: 50 nm.

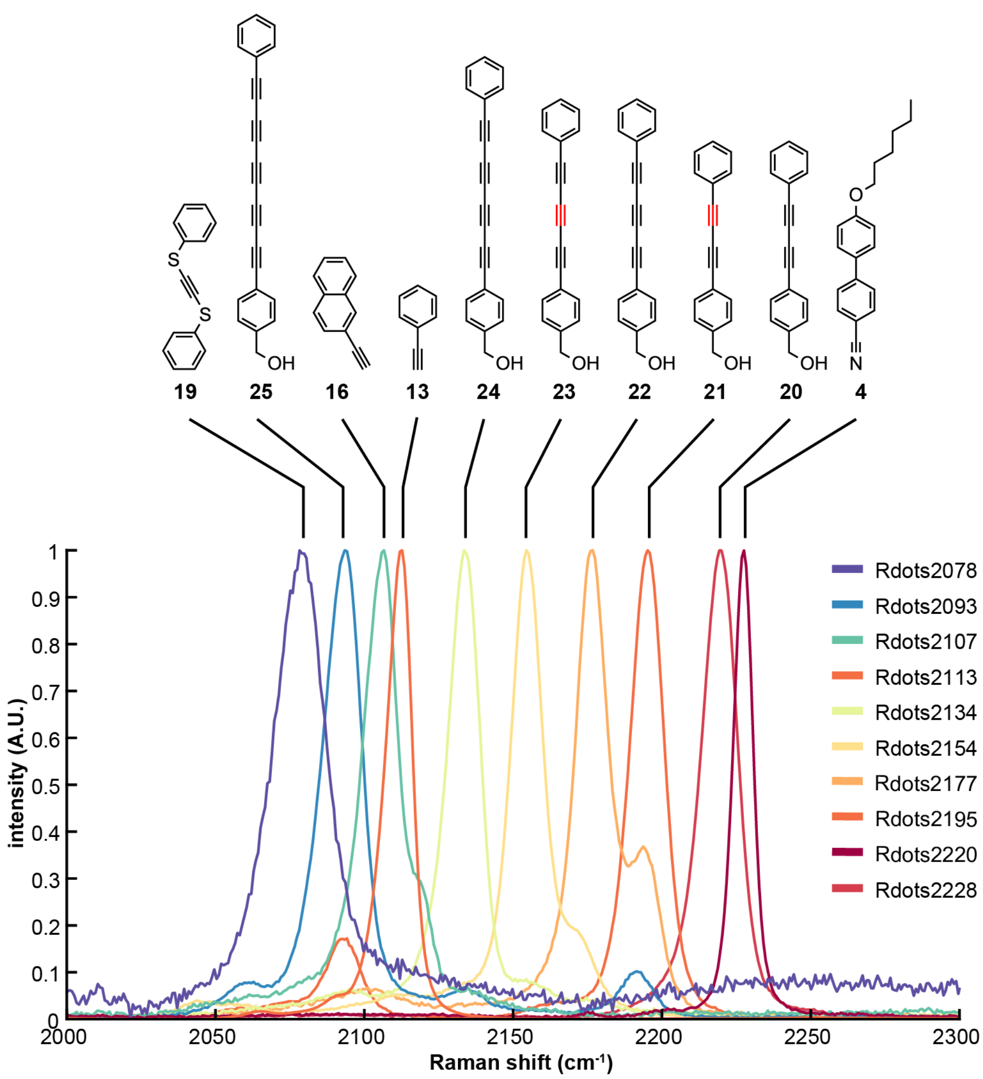

Supplementary Figure 4. 10 Rdots with resolvable Raman peaks. Total 10 Rdots with resolvable Raman peaks were generated with selected Carbow dyes and commonly used Raman probes. A.U.: arbitrary units.

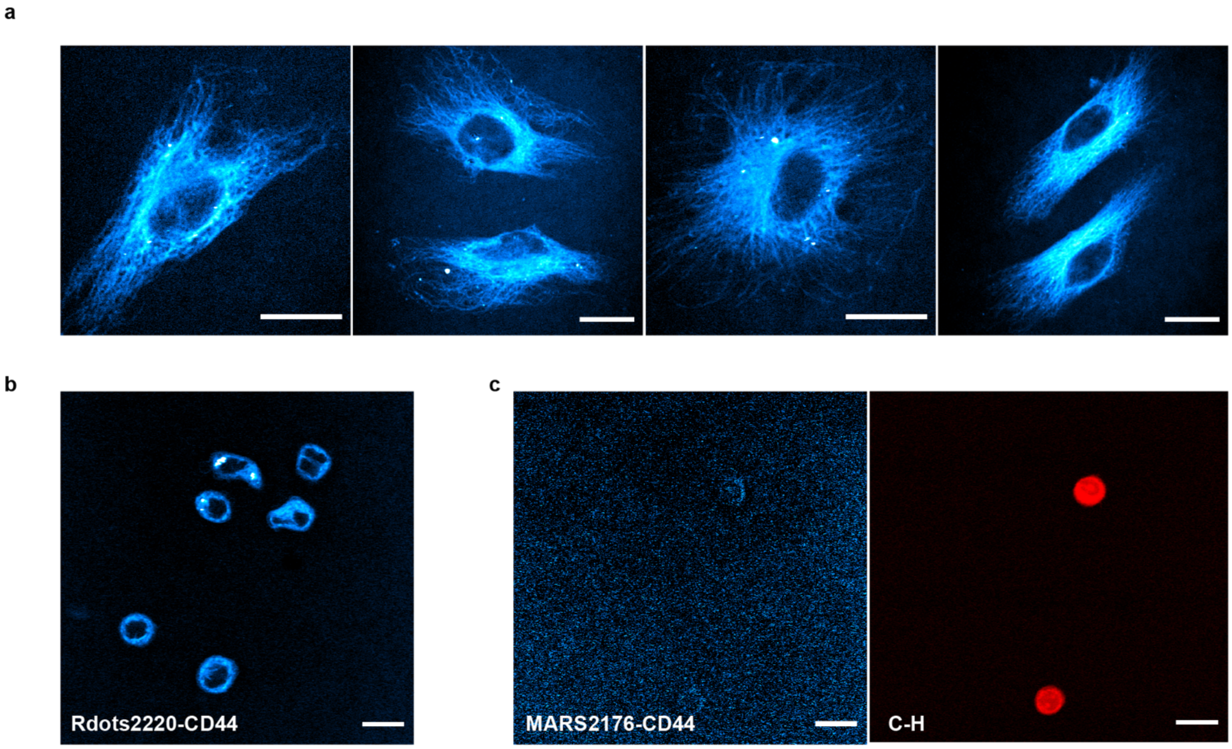

Supplementary Figure 5. Immunostaining with Rdots using only primary antibodies. (a), microtubule staining with Rdots using only primary anti-a-tubulin antibodies. Staphylococcus protein A (SpA) was bioconjugated to Rdots2134, and anti-a-tubulin antibodies was immobilized by the SpA for immunostaining. Each individual of microtubule filaments is clearly visualized, suggesting high sensitivity even without the additional signal amplification of secondary antibodies. Scale bar, 20μm. **(b)**, Low abundant membrane protein (CD44) staining with Rdots using only primary antibodies. Anti-CD44 primary antibodies were directly bioconjugated to Rdots2220 and used for visualizing CD44 in fixed SKBR3 cells. The membrane-distributed signal suggests superb sensitivity even for relatively low abundance membrane proteins, which is not possible by Carbow dyes alone. Scale bar, 20μm. **(c),** CD44 immunostaining with MARS2176. CD44 proteins were first stained with primary antibody and then stained with MARS2176 conjugated secondary antibody. The lack of signal indicates that MARS dyes might not be sensitive enough to visualize low abundant surface markers. The C-H channel indicates the existence of the cells. Scale bar, 20μm.

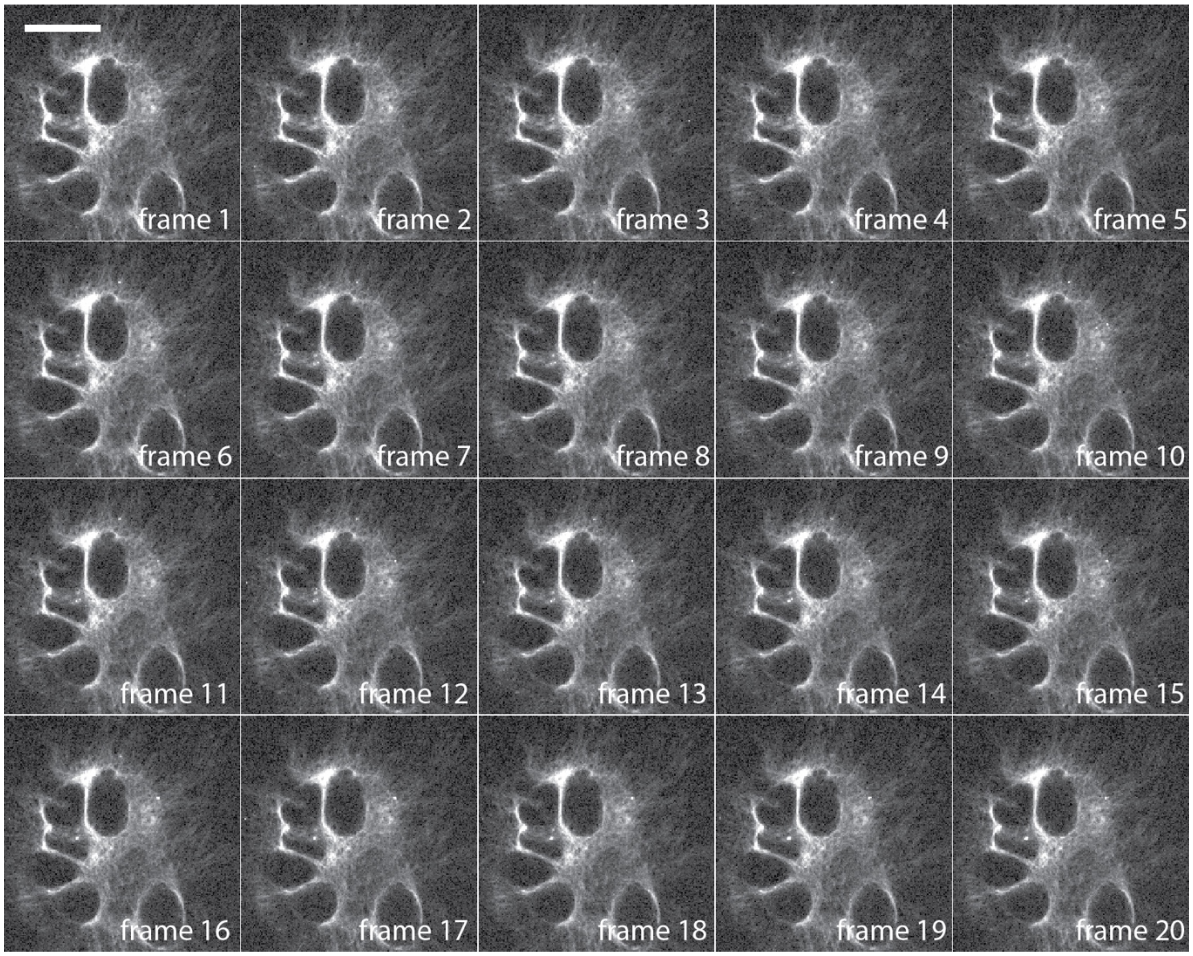

Supplementary Figure 6. Rdots are photo-stable. HeLa cells were first stained with Rdots 2220, and 20 SRS images were taken continuously using the same acquisition parameters as in the other images (left to right, top to bottom). No significant photobleaching was observed even for the last frame, indicating Rdots are photo-stable. This attribute facilitates multiplexed imaging as the last imaged target can still maintain its full intensity. Scale bar, 20μm.

Supplementary note

Estimation of the number of Raman probe molecule per nanoparticle

The number of Raman probe molecule per nanoparticle were estimated with two independent methods.

1) The number of Raman probe molecules can be estimated by dividing the RIE value of Rdots by the RIE value of the Raman probe. For example, RIE(Rdots2220) = 8.1×10^4, and RIE(Carbow 2-yne) = 30, so the number of 2-yne per Rdots2220 is 8.0×10^4 / 30 = 2.7×10^3.

2) The number of Raman probe molecules can also be estimated by using Raman intensity ratio of the Raman probe and polystyrene nanoparticle matrix. This assumes that the density of the polystyrene plate is the same as polystyrene nanoparticles, which is reasonable (1.055g/cm^3 of PS NPs provided by the manufacture's document, and 1.05g/cm^3 of general-purpose polystyrene). We first acquired the Raman spectrum of 100 mM 2-yne DMSO solution (Supplementary Figure 7 A) and of a thick polystyrene plate (Supplementary Figure 7 B) quantitatively by making sure the laser illumination volume was completely filled and the signal intensity did not change while adjusting z-position. Then the Raman signal ratio (R) of 1 mol 2-yne and 1 mol polystyrene (of the C≡C vibration in 2-yne at 2220 cm^{-1} and C–H vibration in styrene at 3055 cm^{-1}, respectively). can be calculated by dividing the two integrated area of the two peaks. The calculation yields $R\approx1.1\times10^2$, which means that Raman signal of 1 mol 2-yne is 1.1×10^2 larger than 1 mol styrene monomer in the polymer (of the C≡C vibration in 2-yne at 2220 cm^{-1} and C–H vibration in styrene at 3055 cm^{-1}, respectively).

   The spectrum of Rdots2220 was then acquired quantitatively (Supplementary Figure 7 C). Similar, the ratio of the two peaks ($R_{2220/3055}$) inside the Rdots is calculated: $R_{2220/3055} \approx 12$. Then the number of styrene monomer in the 20nm nanoparticle can be estimated by:

$$N_{styrene} = \frac{N_A \times V \times \rho}{m}$$

   , where N_A is the Avogadro constant, V is the volume of a 20nm diameter nanoparticle, ρ is the density of polystyrene nanoparticles, and m is the molecular mass of styrene. The calculation yields $N_{styrene} \approx 2.5\times10^4$. The number of 2-yne molecules per Rdots therefore can be found as:

$$N_{2-yne} = \frac{N_{styrene} \times R_{2220/3055}}{R}$$

   Putting in the number acquired previously, $N_{2-yne} \approx 2.7\times10^3$

The numbers of Raman probe molecules within a single Rdots found by the two independent methods are similar, indicating a good consistency.

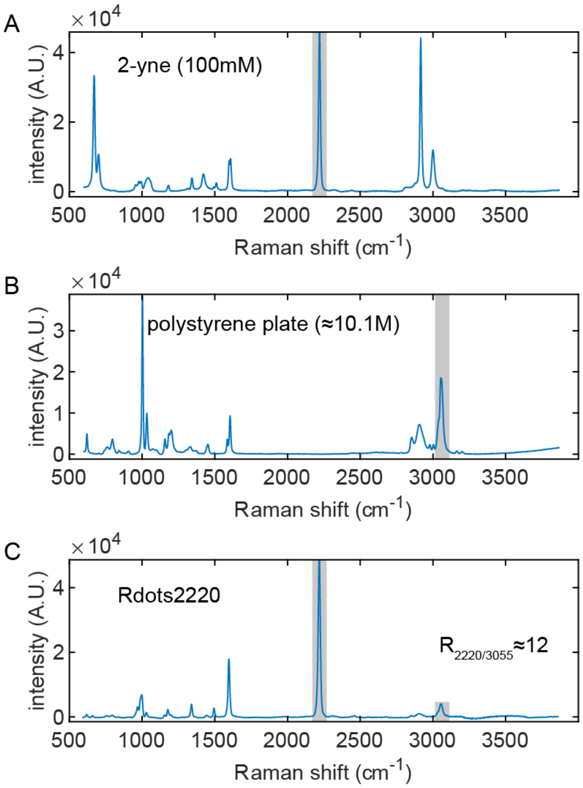

Supplementary Figure 7. Spontaneous Raman spectra of Carbow 2-yne stock solution, polystyrene plate and Rdots2220. The molarity of the styrene monomers in polystyrene was converted from the density of polystyrene (1.05g/cm^3) and molecular mass of styrene (104g/mol). A.U.: arbitrary units.

Estimation of SRS cross sections

The SRS cross section estimation of all the Raman probes mentioned in the article was based on the SRS cross section of EdU and the RIE values of the probes.

It has been previously reported[2] that the SRS cross section of C–O vibration ($\sigma_{SRS,(C–O)}$) at 1030 cm^{-1} in methanol is 1.2×10^{-22} cm^2. We then took spontaneous Raman spectra of both pure methanol and EdU with 532nm excitation wavelength (Supplementary Figure 8). The ratio of the integrated peak area of C≡C vibration in EdU and C–O vibration in methanol was found to be 5.0×10^1, and therefore the SRS cross section of C≡C vibration $\sigma_{SRS,EdU(C\equiv C)} = 1.2 \times 10^{-22}$ $cm^2 \times 5.0 \times 10^1 = 6.0 \times 10^{-21}$ cm^2. The SRS cross section of C≡C vibration and the C≡N vibration can be roughly estimated by $\sigma_{SRS}= \sigma_{SRS,EdU(C\equiv C)} \times$RIE. For example, $\sigma_{SRS,2\text{-}yne(C\equiv C)} = \sigma_{SRS,EdU(C\equiv C)} \times RIE_{2\text{-}yne} = 6.0 \times 10^{-21}$ $cm^2 \times 8.1 \times 10^4 \approx 4.9\times 10^{-16}$ cm^2. The RIE values and the SRS cross section of Raman probes used in the study can be found in Supplementary Table 1.

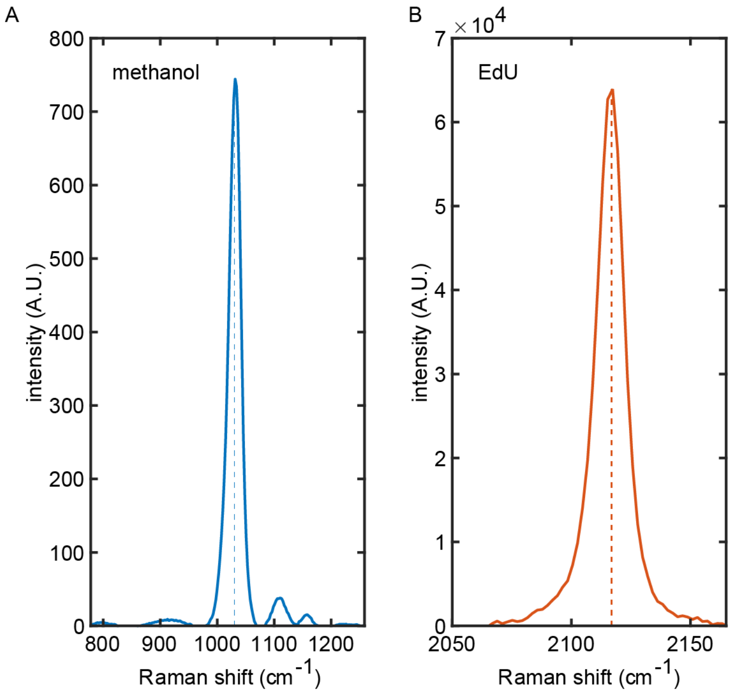

Supplementary Figure 8. Normalized spontaneous Raman spectra of pure methanol (~24.4M) and EdU (20mM). The SRS cross section of EdU was extrapolated based on the intensity difference and the reported SRS cross section of C–O vibration at 1030 cm^{-1} in methanol. A.U.: arbitrary units.

Supplementary tables

Supplementary Table 1. Summary of properties of Raman probes and Rdots in the study

	Compound	RIE (of probes)	RIE (of Rdots)	$\sigma_{SRS,(C\equiv C) or (C\equiv N)}$ (of Rdots)*	# of probes per NP*
1	4-formylbenzonitrile	0.13	9.4E+02	5.6E-18	7.1E+03
2	4-acetylbenzonitrile	0.17	1.9E+03	1.1E-17	1.1E+04
3	4-4-biphenyldicarbonitrile	0.48	7.4E+02	4.4E-18	1.5E+03
4	4-(3-hydroxyphenyl)benzonitrile	0.20	1.5E+03	9.2E-18	7.8E+03
5	benzonitrile	0.09	2.1E+03	1.2E-17	2.2E+04
6	4-(Hexyloxy)-4-biphenylcarbonitrile	0.32	7.3E+03	4.4E-17	2.3E+04
7	4-proproxy-biphenyl-4-carbonitrile	0.33	8.2E+03	4.9E-17	2.5E+04
8	4-(bromoethynyl)-1,1'-biphenyl	1.57	1.3E+04	7.8E-17	8.3E+03
9	1-phenyl-2-(trimethylsilyl)acetylene	1.13	1.9E+04	1.1E-16	1.7E+04
10	4,4'-bis((trimethylsilyl)ethynyl)-1,1'-biphenyl	4.41	1.2E+04	7.5E-17	2.8E+03
11	2-trimethylsilyl-ethynyl-9H-fluorene	1.08	2.4E+04	1.5E-16	2.2E+04
12	2,7-bis((trimethylsilyl)ethynyl)-9H-fluorene	4.46	1.6E+04	9.8E-17	3.7E+03
13	phenylacetylene	0.49	4.2E+03	2.5E-17	8.5E+03
14	4-ethynylbenzaldehyde	1.03	3.1E+03	1.9E-17	3.1E+03
15	4-ethynylbiphenyl	1.03	9.4E+03	5.7E-17	9.2E+03
16	2-ethynyl-naphthalene	0.78	1.3E+04	7.9E-17	1.7E+04
17	2-ethynyltoluene	0.59	1.1E+04	6.6E-17	1.9E+04
18	2,6-diethynylnaphthalene	2.25	6.0E+03	3.6E-17	2.7E+03
19	1,2-bis(phenylthio)ethyne	FL	5.9E+03	3.5E-17	N/A
20	Carbow 2-yne	30	8.1E+04	4.9E-16	2.7E+03
21	Carbow 2-yne-13C	same as **20**	same as **20**	same as **20**	same as **20**
22	Carbow 3-yne	92	4.2E+04	2.5E-16	4.5E+02
23	Carbow 3-yne-13C	same as **22**	same as **22**	same as **22**	same as **22**
24	Carbow 4-yne	200	5.9E+04	3.5E-16	2.9E+02
25	Carbow 5-yne	380	3.1E+04	1.9E-16	8.3E+01

* These values were roughly estimated by RIE values mentioned in the SI text.

Supplementary Table 2. Structures of 25 Raman probes used to generate Rdots.

**1**	4-formylbenzonitrile	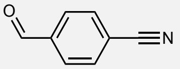
**2**	4-acetylbenzonitrile	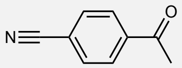
**3**	4,4′-biphenyldicarbonitrile	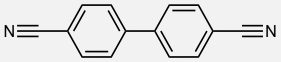
**4**	4-(3-hydroxyphenyl) benzonitrile	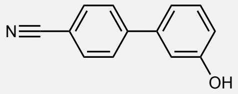
**5**	benzonitrile	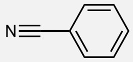
**6**	4'-(hexyloxy)-4-biphenylcarbonitrile	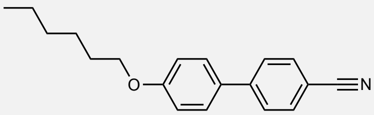
**7**	4'-propoxy-biphenyl-4-carbonitrile	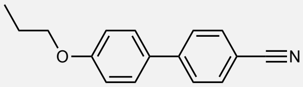
**8**	4-(bromoethynyl)-1,1'-biphenyl	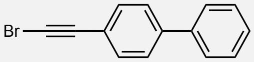
**9**	1-phenyl-2-(trimethylsilyl)acetylene	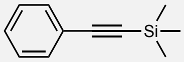
**10**	4,4'-bis((trimethylsilyl) ethynyl)-1,1'-biphenyl	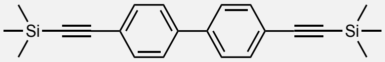
**11**	((9*H*-fluoren-2-yl)ethynyl)trimethylsilane	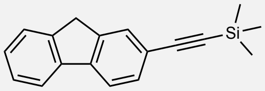
**12**	2,7-bis((trimethylsilyl) ethynyl)-9*H*-fluorene	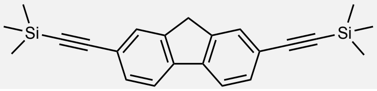

**13**	phenylacetylene	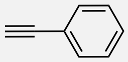
**14**	4-ethynylbenzaldehyde	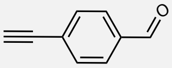
**15**	4-ethynylbiphenyl	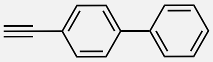
**16**	2-ethynyl-naphthalene	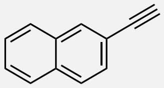
**17**	2-ethynyltoluene	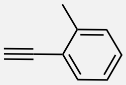
**18**	2,6-diethynylnaphthalene	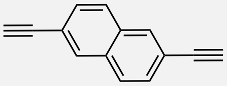
**19**	1,2-bis(phenylthio)ethyne	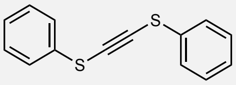
**20**	Carbow 2-yne	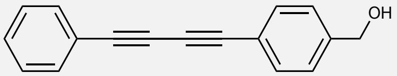
**21**	Carbow 2-yne-13C	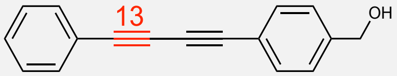
**22**	Carbow 3-yne	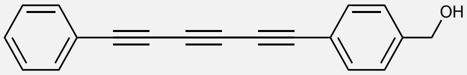
**23**	Carbow 3-yne-13C	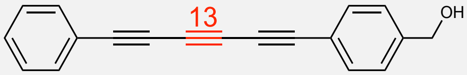
**24**	Carbow 4-yne	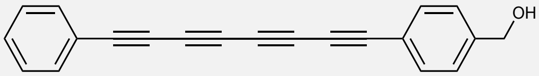
**25**	Carbow 5-yne	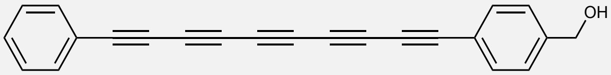

Supplementary Table 3. Concentration of antibodies used in immunostaining

Target	Manufacturer	Cat. #	Staining conc. (ug/ml)	Dilution
a-tubulin	Invitrogen	62204	2	1:100
vimentin	Cell Signaling Technology	5741	0.45	1:100
CD44	Invitrogen	14044185	N/A	N/A
e-cadherin	Cell Signaling Technology	3195	0.52	1:100
mouse IgG	Jackson ImmunoResearch	115-005-146	N/A	N/A
rabbit IgG	Jackson ImmunoResearch	111-005-144	N/A	N/A

Supplementary Table 4. Concentration of Carbow dyes to generate Rdots

Dye	Final concentration (mM)	Stock concentration (mM)
Carbow 2ynes*	15	300
Carbow 3ynes*	2.5	30
Carbow 4yne	1	10
Carbow 5yne	1	10

* The isotope versions were used at the same concentrations.

Supplementary references

1. Yamakoshi H*, et al.* Alkyne-tag Raman imaging for visualization of mobile small molecules in live cells. *J Am Chem Soc* **134**, 20681-20689 (2012).

2. Wei L, Min W. Electronic Preresonance Stimulated Raman Scattering Microscopy. *J Phys Chem Lett* **9**, 4294-4301 (2018).
