# Supplementary material for: Ultra-bright Raman dots for multiplexed optical imaging
Source: Nat Commun. 2021 Feb 26;12:1305. doi: 10.1038/s41467-021-21570-0 (PMC7910594; doi:10.1038/s41467-021-21570-0)
Supplement: Supplementary file 3 — Reporting Summary [file 41467_2021_21570_MOESM3_ESM.pdf]

nature research

Corresponding author(s): Dr. Wei Min

Last updated by author(s): Jan 7, 2021

Reporting Summary

Nature Research wishes to improve the reproducibility of the work that we publish. This form provides structure for consistency and transparency in reporting. For further information on Nature Research policies, see our Editorial Policies and the Editorial Policy Checklist.

Statistics

For all statistical analyses, confirm that the following items are present in the figure legend, table legend, main text, or Methods section.

n/a	Confirmed	
☐	☒	The exact sample size (n) for each experimental group/condition, given as a discrete number and unit of measurement
☐	☒	A statement on whether measurements were taken from distinct samples or whether the same sample was measured repeatedly
☐	☒	The statistical test(s) used AND whether they are one- or two-sided<br>*Only common tests should be described solely by name; describe more complex techniques in the Methods section.*
☐	☒	A description of all covariates tested
☐	☒	A description of any assumptions or corrections, such as tests of normality and adjustment for multiple comparisons
☐	☒	A full description of the statistical parameters including central tendency (e.g. means) or other basic estimates (e.g. regression coefficient) AND variation (e.g. standard deviation) or associated estimates of uncertainty (e.g. confidence intervals)
☐	☒	For null hypothesis testing, the test statistic (e.g. F, t, r) with confidence intervals, effect sizes, degrees of freedom and P value noted<br>*Give P values as exact values whenever suitable.*
☒	☐	For Bayesian analysis, information on the choice of priors and Markov chain Monte Carlo settings
☒	☐	For hierarchical and complex designs, identification of the appropriate level for tests and full reporting of outcomes
☐	☒	Estimates of effect sizes (e.g. Cohen's d, Pearson's r), indicating how they were calculated

Our web collection on statistics for biologists contains articles on many of the points above.

Software and code

Policy information about availability of computer code

Data collection	Data was collected with Olympus confocal microscopy software package FV3000.
Data analysis	Imaging data was analyzed with ImageJ (v2.1.0), matlab (R2018a) was used for data visualization.

For manuscripts utilizing custom algorithms or software that are central to the research but not yet described in published literature, software must be made available to editors and reviewers. We strongly encourage code deposition in a community repository (e.g. GitHub). See the Nature Research guidelines for submitting code & software for further information.

Data

Policy information about availability of data

All manuscripts must include a data availability statement. This statement should provide the following information, where applicable:
- Accession codes, unique identifiers, or web links for publicly available datasets
- A list of figures that have associated raw data
- A description of any restrictions on data availability

The data that supports this study is available from the corresponding author upon reasonable request.

Field-specific reporting

Life sciences study design

All studies must disclose on these points even when the disclosure is negative.

Sample size	No sample size calculation was performed. For Rdots preparation and characterization, the procedure was repeated more than 30 times as we routinely make new batches for downstream application experiments.<br><br>For representative images shown in Fig. 4a~d, a total of 41 biological replicates with 5 technical replicates for each biological replicate were taken as a routine quality control of Rdots preparation. For representative images shown in Fig. 4e~t, 3 biological replicates with 8 technical replicates for each biological replicate were performed with consistent results. For representative images shown in Fig. 4u~w, a total of 11 biological replicates with 20 technical replicates for each biological replicate were taken as a routine quality control of Rdots preparation. For representative images shown in Fig. 5, 3 biological replicates with 10 technical replicates for each biological replicate were performed with consistent results.<br><br>Since the article focuses more on imaging rather than quantitative analysis, such reproducibility is sufficient to ensure other groups to repeat the results.
Data exclusions	No data was excluded.
Replication	The number of replicates for each individual experiment is indicated in the manuscripts. All replications were successful.
Randomization	We do not have statistical model presented and no randomization is needed in the article.
Blinding	We do not have statistical model presented and no blinding is needed in the article.

Reporting for specific materials, systems and methods

We require information from authors about some types of materials, experimental systems and methods used in many studies. Here, indicate whether each material, system or method listed is relevant to your study. If you are not sure if a list item applies to your research, read the appropriate section before selecting a response.

Materials & experimental systems

n/a	Involved in the study	
☐	☒	Antibodies
☐	☒	Eukaryotic cell lines
☒	☐	Palaeontology and archaeology
☒	☐	Animals and other organisms
☒	☐	Human research participants
☒	☐	Clinical data
☒	☐	Dual use research of concern

Methods

n/a	Involved in the study	
☒	☐	ChIP-seq
☒	☐	Flow cytometry
☒	☐	MRI-based neuroimaging

Antibodies

Antibodies used	Invitrogen 62204; Invitrogen 14044185; CST 5741; CST 3195; JacksonImmunoResearch 115-005-146 and 111-005-144
Validation	Antibodies were validated by the manufacturers.<br><br>According to the manufacturer, Invitrogen 62204 has been successfully used in Western blot, immunohistochemistry, immunoprecipitation, electron microscopy, immunocytochemistry and immunofluorescent applications.<br><br>According to the manufacturer, Invitrogen 14044185 has been reported for use in flow cytometric analysis, immunoprecipitation, immunohistochemical staining and immunoblotting (non-reducing conditions).<br><br>According to the manufacturer, CST5741 detects endogenous levels of total vimentin protein.<br><br>According to the manufacturer, CST3195 detects endogenous levels of total E-cadherin protein. The antibody does not cross-react with related family members, such as N-cadherin.

Eukaryotic cell lines

Policy information about cell lines

Cell line source(s)	Cos7, HeLa and SKBR3 were purchased from ATCC.

Authentication	Cells were authenticated by ATCC.
Mycoplasma contamination	Cells were not tested for Mycoplasma
Commonly misidentified lines (See ICLAC register)	None
